# Periodontal and Systemic Diseases: A Descriptive Analysis of Awareness Among the General Saudi Population

**DOI:** 10.7759/cureus.56088

**Published:** 2024-03-13

**Authors:** Muzammil Moin Ahmed, Osama Saleh H Altuwayjiri

**Affiliations:** 1 Department of Dental and Oral Health, College of Applied Health Sciences in Ar Rass, Qassim University, Al Qassim, SAU

**Keywords:** descriptive analysis, systemic diseases, periodontal diseases, awareness, periodontal-systemic diseases

## Abstract

Background

Taking into account the limited availability of research data, this study aimed to determine the general Saudi population’s awareness of the link between periodontal diseases and systemic diseases.

Methodology

A structured online questionnaire with eight awareness items, apart from demographic variables, was distributed through email, WhatsApp, and Telegram to a sample of 500 individuals. The data were analyzed using a simple descriptive statistical approach and interpreted as ratios for comparison. The awareness regarding systemic diseases associated with periodontal diseases was classified into the following four categories based on the Bloom cutoff points: high (>80%), average (60-79%), low (40-59%), and extremely low (<40%).

Results

A response rate of 68% was reached with the participation of 340 Saudi citizens residing in the Al Qassim region. Overall, 61.22% of research participants had an average awareness of the link between periodontal and systemic diseases. Almost two-thirds (>60%) of participants were aware that periodontal diseases and systemic diseases have an association and that individuals with systemic diseases need a periodontal checkup. A majority (85%) of participants opined that periodontal treatment has the potential to enhance overall health. Nonetheless, only a few participants (60%) were aware of the association between diabetes mellitus and periodontal diseases, and they had a limited awareness of the association with other systemic diseases.

Conclusions

Although the Saudi general population possesses average awareness about the relationship between periodontal diseases and systemic diseases, their awareness about different systemic diseases and conditions is extremely low, particularly regarding infertility, stroke, and metabolic diseases. The present research indicates a deficiency in the efforts by healthcare professionals, community service providers, and community administrators to educate the general public regarding the association between periodontal diseases and systemic diseases. This awareness is crucial for individuals to control these intricate, interconnected diseases.

## Introduction

The human body, with all of its organs and tissues, functions as a cohesive entity rather than as independent units. It is often believed that periodontal diseases only affect the periodontium, and their influence is contained in the oral cavity. As periodontal diseases rarely cause pain or any other acute symptoms, this belief is further strengthened. It is becoming more and more clear that periodontal diseases go much beyond what is generally believed, as a result of the advancement of scientific research and technological developments. Periodontal pathogens and their products can get through the epithelial barrier as well as other barriers, such as the blood-brain barrier, and make their way into the bloodstream and other tissues [1,2]. Such infiltration can occur at any point in the progression of periodontal disease, as well as during routine teeth cleaning and periodontal procedures [3-5]. Periodontal infections and their virulence factors have the potential to stimulate immunological responses from the host and to elicit systemic inflammation, which itself is accompanied by the participation of immune cells, cytokines, and chemokines [6-8]. Periodontal bacterial infiltration and elicitation of host immune responses impact a broad spectrum of systemic diseases and conditions. Scientific literature indicates a linkage of periodontal diseases with diabetes mellitus, cardiovascular diseases, hepatorenal disorders, adverse pregnancy outcomes, cognitive impairment, Alzheimer’s disease, cancers, and many more [9-16]. A few of these conditions are thought to have a bilateral relationship with periodontal diseases and have been identified in the most recent classification of periodontal and peri-implant diseases [17].

As a result of these implications, it is crucial for the general population as well as those working in the health profession to be informed about the association that exists between periodontal and systemic diseases. Information about awareness can help bring people’s attention to this significant problem, as well as take corrective steps to improve awareness if needed. Numerous studies have been conducted to evaluate the level of knowledge of this issue among dental students and professionals [18-22]. There have only been a few studies on the comprehension of this link among the general community, particularly in the Saudi population [20,23]. Therefore, and taking into account the limited availability of research data, this study was conducted to determine the level of awareness about the association between periodontal diseases and systemic diseases among the general Saudi population.

## Materials and methods

This cross-sectional survey was conducted by the final-year students and faculty as part of the mandatory research activity of the department, with the permission of the college ethical clearance committee (reference number: IEC/QA/21.08.98). The survey was conducted among a sample of 340 Saudi citizens who consented to participate in the study after explaining to them the purpose of the study.

The study included individuals who met specific criteria to ensure the integrity and relevance of the research findings. First, participants needed to hold Saudi citizenship, thereby aligning with the study’s focus on the Saudi population. Additionally, individuals residing in the Al Qassim region were targeted for inclusion, aiming to capture a localized perspective and mitigate potential regional variations in awareness and healthcare access. Voluntary participation was another crucial criterion, with participants required to provide informed consent indicating their willingness to contribute to the study. Moreover, participants were expected to have a basic understanding of the study’s purpose, ensuring that their decision to participate was informed and based on a clear comprehension of the research objectives.

Conversely, some individuals were excluded from participation to uphold the study’s rigor and validity. Non-Saudi citizens were excluded to maintain the study’s focus on the target population and minimize potential confounding factors stemming from cultural or socioeconomic differences. Additionally, individuals working or studying dentistry or medicine were excluded to prevent bias resulting from their specialized knowledge, which could skew their awareness of the association between periodontal and systemic diseases. Furthermore, individuals unable to provide informed consent due to factors such as cognitive impairment or language barriers were excluded to ensure that participation was based on voluntary and well-informed decisions, adhering to ethical standards. These inclusion and exclusion criteria were established to create a representative sample of the target population while minimizing sources of bias that could impact the study’s outcomes.

The sample size was estimated using the data obtained from a previous study conducted by Alsalleeh et al. [23]. For the expected prevalence of 59.8%, the required sample size was 299 for a relative precision of ±10% in estimating the prevalence with a 95% confidence interval of 49.8-69.8% and considering a 10% attrition/loss.

This survey was conducted using a self-administered structured questionnaire. Face validity of the questionnaire was assessed using five expert consensuses. Experts evaluated each item for its framing and grammar, understandability, and relatedness, as well as for any suggestions. The questionnaires were collected and suggestions were considered. The questionnaire consisted of two sections. The first section included three items about the age of the participants, gender, and education of the participants. The second section included eight items with predetermined answers designed to gauge respondents’ levels of awareness of the link between systemic and periodontal diseases. The items included questions on the association between systemic health and periodontal health, the association between periodontal health and systemic health, the association between systemic disease and periodontal disease, the influence of the medication on periodontal health, and specific systemic conditions affecting periodontal health.

The data were analyzed using a simple descriptive statistical approach and interpreted in ratios for comparison. The awareness regarding systemic diseases associated with periodontal diseases was classified into the following four categories based on the cutoff points suggested by Bloom: high (>80%), average (60-79%), low (40-59%), and extremely low (<40%) [24].

## Results

The questionnaire was distributed to 500 Saudi individuals in the Al Qassim region, of whom 340 responded, for a response rate of 68%. The characteristics of the participants are provided in Table 1. Males (n = 239; 70.3%) and people in late adolescence (n = 128; 37.65%) had greater participation rates in this research. More than half of the participants, i.e., 196 (57.65%) had a bachelor’s degree.

**Table 1:** Demographic characteristics of the study participants.

	N	Ratio (%)
Age (years)		
Early adolescence (12–16)	6	1.76
Late adolescence (17–25)	128	37.65
Young adult (26–35)	69	20.3
Late adult (36–45)	77	22.65
Early elderly (46–55)	46	13.53
Late elderly (56–65)	12	3.53
Senior (66 and above)	2	0.58
Total	340	100
Gender		
Male	239	70.3
Female	101	29.7
Total	340	100
Education		
Never gone to school	1	0.29
Till 7th	11	3.23
Till 10th	70	20.59
Diploma	35	10.3
Bachelor’s degree	196	57.65
Master’s degree	17	5
Doctoral degree	10	2.94
Total	340	100

About 230 (67.65%) research participants had an average awareness of the link between periodontal and systemic diseases. The response ratios of research participants to eight questionnaire items are shown in Table 2. Only 132 (38.82%) participants were aware that periodontal diseases and systemic diseases have an association, and around 205 (60.3%) individuals were aware that patients suffering from systemic diseases need periodontal check-ups. About 289 (85%) participants opined that periodontal treatment has the potential to enhance overall health. Only 125 (36.76%) participants were aware of the association between diabetes mellitus and periodontal diseases, and they had a limited awareness of the association with other systemic diseases, as indicated in Table 2.

**Table 2:** Participants’ awareness about the association between periodontal and systemic diseases.

Awareness questions	Option	N	%	Ratio interpretation
Do you think systemic health affects periodontal health?	Yes	213	62.64	Average
	No	39	11.47	
	Don’t know	88	25.89	
Do you think periodontal health affects systemic health?	Yes	230	67.65	Average
	No	65	19.12	
	Don’t know	45	13.23	
Have you ever heard or been aware of any systemic diseases associated with periodontal diseases?	Yes	132	38.82	Extremely low
	No	106	31.18	
	Don’t know	102	30	
If you answered yes to the above question, then which diseases have you heard or been aware of? (open-ended question)	Diabetes mellitus	60	17.64	Extremely low
	Hypertension	7	2.05	Extremely low
	Heart diseases	5	1.47	Extremely low
	Renal failure	1	0.29	Extremely low
	Preterm labor	1	0.29	Extremely low
	Osteoporosis	1	0.29	Extremely low
Do you think medications have an influence on periodontal health?	Yes	180	52.94	Low
	No	74	21.76	
	Don’t know	86	25.3	
Do you think patients suffering from systemic diseases need periodontal check-ups?	Yes	205	60.3	Average
	No	69	20.3	
	Don’t know	66	19.4	
Do you think periodontal treatment can improve general health?	Yes	289	85	High
	No	20	5.89	
	Don’t know	31	9.11	
Which of the below diseases do you think are associated with periodontal diseases?	Heart diseases	92	27.06	Extremely low
	Respiratory diseases	73	21.47	Extremely low
	Liver diseases	97	28.53	Extremely low
	Kidney diseases	53	15.59	Extremely low
	Diabetes mellitus	125	36.76	Extremely low
	Adverse pregnancy outcomes	39	11.47	Extremely low
	Hypertension	47	13.82	Extremely low
	Cancer	66	19.41	Extremely low
	Metabolic diseases	27	7.94	Extremely low
	Infertility	3	0.88	Extremely low
	Stroke	7	2.06	Extremely low
	Rheumatoid arthritis	35	10.29	Extremely low
	Obesity	39	11.47	Extremely low
	Cognitive impairment	12	3.52	Extremely low
	Mental disorders	46	13.53	Extremely low
	Blood disorders	98	28.82	Extremely low

**Figure 1:**
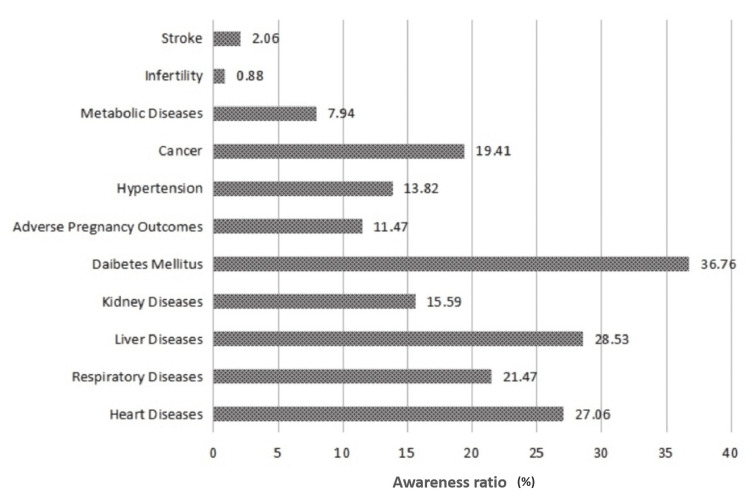
Awareness ratio of periodontal diseases relative to systemic diseases.

When awareness ratios about various systemic illnesses and their association with periodontal diseases were categorized, the awareness of respondents was deemed extremely low. The awareness ratio of periodontal diseases relative to various systemic diseases is depicted in Figure 1. The awareness ratio for the relationship between periodontal diseases and infertility was the lowest (N = 3; 0.88%). Similarly, the awareness ratios for the association between periodontal diseases and stroke and metabolic diseases were 2.06% (n = 7) and 7.94% (n = 27), respectively.

## Discussion

The repercussions of periodontal diseases extend beyond the oral cavity, as periodontal inflammation and pathogens impact a broad spectrum of immunoinflammatory components, directly or indirectly affecting several organs and systems in the body [25]. It is crucial for the general populace to be aware of these ramifications. The levels of awareness that dental and medical experts have regarding the relationship between periodontal disease and systemic diseases are highly evident, as evidenced by recent research concerning this topic. Regrettably, the extent of awareness regarding this association among the broader Saudi populace remains uncertain, given the scarcity of published research on the subject. Therefore, this study intended to assess the level of awareness regarding the relationship between periodontal and systemic diseases in the Saudi population.

The key outcome of the research is the average level of awareness of the relationship between periodontal and systemic diseases among the general population in Saudi Arabia. The awareness ratio identified in this study (61.22%) is comparatively higher than the one found in the research by Alsalleeh et al. (50%) [23]. This disparity could be traced to Alsalleeh et al.’s study, which incorporated participants from five distinct regions, whereas this study only collected data from the Qassim region of Saudi Arabia. Another reason for the disparity between the results of the two studies could be that Alsalleeh et al. recruited both Saudi residents and Arabic-speaking adults in their research, whereas, in the current study, only Saudi citizens were included, with the exclusion of Saudi residents of other nationalities [26]. The level of awareness identified in this study is also higher than the findings reported by Alessa et al. (52.3%) [26]. The main factor contributing to this difference may be the recruitment criteria, which restricted the educational level of participants to high school, as well as the smaller sample size in Alessa et al.’s study [26].

Another finding of the present research is that the Saudi general population has extremely low awareness of the numerous systemic diseases connected with periodontal diseases, despite their agreement on the potential impact of systemic and periodontal diseases on one another. This study investigated the awareness of periodontal diseases related to 11 systemic diseases that are currently recognized by scientific evidence, in contrast to the studies of Alsalleeh et al. and Alessa et al., which considered only four and six systemic diseases, respectively [23,26]. The present study’s findings indicate diabetes mellitus is slightly more acknowledged by the general population compared to other systemic diseases to be associated with periodontal diseases; however, this awareness was extremely low. Bahammam et al. also reported a similar finding, indicating limited awareness regarding the relationship between diabetes mellitus and periodontal disease [27]. The lowest level of awareness was observed regarding infertility, stroke, and metabolic diseases. A study conducted by Tao et al. revealed an association between periodontal disorders and male infertility [28], while another study by Ricci et al. indicated a similar link between periodontal diseases and female infertility [29]. A systematic review by Fagundes et al. concluded that periodontal diseases may pose an increased risk of ischemic stroke incidence [30]. It is surprising that, despite the abundance of scientific literature, there is a low level of awareness about the association of periodontal diseases with metabolic diseases. Pirih et al. not only observed this link but also hypothesized that metabolic diseases could impact the oral microbiome [31].

While this study provided further insights into the awareness of the association between periodontal and systemic diseases in the general Saudi population, it is important to acknowledge a limitation. The study is limited by its inability to assess awareness about research variables such as age, gender, education, and so on. However, this limitation does not have a substantial impact on the primary outcomes of this research.

The present research indicates a deficiency in the efforts by healthcare professionals, community service providers, and community administrators to educate the general public regarding the association between periodontal diseases and systemic diseases. This awareness is crucial for individuals to control these intricate, interconnected diseases.

## Conclusions

Although the Saudi general population possesses average awareness of the relationship between periodontal diseases and systemic diseases, their awareness of different systemic diseases and conditions is extremely low, particularly for infertility, stroke, and metabolic diseases.
